# A Case of Wellens Syndrome in a 30-Year-Old Woman From Sub-Saharan Africa: A Perplexing Clinical Entity With Invaluable Lessons

**DOI:** 10.1177/2324709620918552

**Published:** 2020-05-05

**Authors:** Pedro Pallangyo, Smita Bhalia, George Longopa, Kawajika Mwinyipembe, Jalack Millinga, Nsajigwa Misidai, Happiness Judical Swai, Zabella Seif Mkojera, Naairah Rashid Hemed, Rydiness Mulashani, Polycarp Seraphine, Regan Valerian Massawe, Alice Kaijage, Peter Kisenge, Mohamed Janabi

**Affiliations:** 1 Jakaya Kikwete Cardiac Institute, Dar es Salaam, Tanzania

**Keywords:** Wellens syndrome, LAD coronary T-wave syndrome, LAD stenosis, unstable angina, preinfarction stage, anterior myocardial infarctions, biphasic T-wave inversions

## Abstract

With an estimated contribution of up to 6% of all acute coronary events, young adults are experiencing an escalating burden and mortality attributable to coronary artery disease (CAD) worldwide. Wellens syndrome, a preinfarction clinical entity with distinctive electrocardiographic (ECG) features and high propensity for extensive anterior wall infarctions, affects about 15% of unstable angina subpopulation. We report challenges and lessons learnt from the first ever documented case of Wellens syndrome in Tanzania. A 30-year-old female of African descent was referred to us from an upcountry zonal referral hospital for etiological determination of chest pain and expert management. Her medical history was unremarkable, and she had no apparent risk factors for CAD. She presented with a 7-day history of ongoing sharp central chest pain that was radiating to the neck and jaws and relieved momentarily by morphine. She had stable vitals with an unremarkable systemic examination; however, a 12-lead ECG revealed deeply inverted T-waves on leads V2 through V4. Based on our extensive history and physical examination we came up with a diagnosis of type B Wellens syndrome with impending anterior wall myocardial infarction. She underwent cardiac catheterization that revealed a nonthrombogenic severe subocclusive (>95%) mid left anterior descending artery stenosis. Subsequently, angioplasty was performed successfully with a resolute integrity stent, and TIMI III flow was achieved. To conclude, despite its relative frequency, physicians’ awareness of Wellens syndrome pathognomonic ECG features is of paramount importance to curb its attributable morbidity and mortality.

## Introduction

Since its initial description nearly 4 decades ago,^1^ Wellens syndrome, a relatively common and potentially fatal clinical entity, continues to be underrecognized and lately recognized worldwide.^2-4^ Also referred to as left anterior descending (LAD) coronary T-wave syndrome, this preinfarction stage is characterized by a distinctive electrocardiographic (ECG) pattern (ie, biphasic T-wave inversions on precordial leads) suggestive of a critical stenosis in the LAD coronary artery.^1-5^ It is estimated to affect about 10% to 15% of persons with unstable angina,^4,5^ three quarters of whom will develop extensive anterior wall myocardial infarctions (MIs) if not revascularized timely.^6,7^ Owing to its high attributable morbidity and mortality, Wellens syndrome carries a significant diagnostic and prognostic value.

Wellens syndrome affects all age groups and shares the traditional risk factors with coronary artery disease (CAD), that is, smoking, obesity, dyslipidemia, hypertension, diabetes mellitus, metabolic syndrome, and family history of premature CAD.^8^ Although the diagnosis of Wellens requires a noninvasive rather straightforward ECG criteria, this clinical entity often poses a diagnostic challenge to physicians all over the globe. The dilemma in recognizing this portentous syndrome lies in (1) inadequate physician skills in ECG interpretation, (2) frequent absence of clear ischemia at initial evaluation, (3) frequent manifestation of the signs during pain-free intervals, and (4) the sharing of the characteristic ECG pattern with several other common clinical conditions including hypokalemia, intracerebral hemorrhage, persistent juvenile T-wave inversion, pulmonary embolism, right bundle branch block, or at times it may be just a normal variant.^1-9^ With the increasing global prevalence of CAD risk factors in younger populations,^10^ an increase in presentation of Wellens syndrome at earlier age is foreseeable. We report the experience and lessons learnt from the first ever documented local case of Wellens syndrome in a 30-year-old woman of African origin from northern Tanzania.

## Case Description

**Figure 1.**
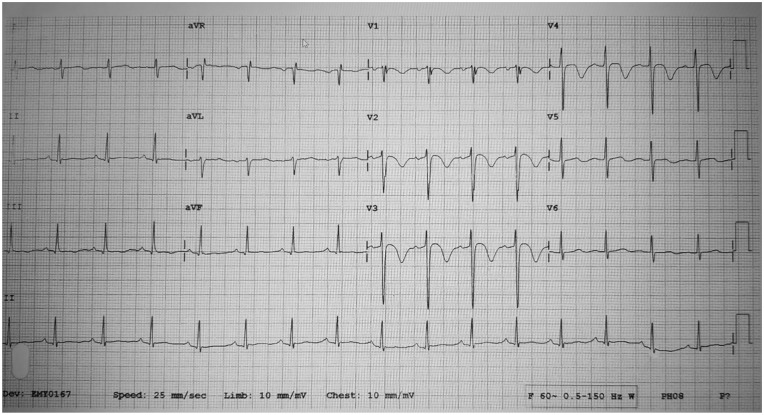
A 12-lead ECG showing strain pattern.

A 30-year-old female primary school teacher of African descent was referred to Jakaya Kikwete Cardiac Institute from a northern zone referral hospital (540 km away) for etiological determination of chest pain and expert management. She has never smoked cigarettes or used illicit drugs and denied a family history of heart disease. Her medical history was only significant for menorrhagia. She presented to us with a 7-day history of ongoing sharp central chest pain that was radiating to the neck and jaws and relieved temporarily by morphine. The pain woke the patient from sleep and was accompanied by shortness of breath, diaphoresis, and nausea without emesis. She was hospitalized at the upcountry hospital for the initial 3 days of chest pain and was prescribed acetylsalicylic acid 75 mg od (once a day), clopidogrel 75 mg od, atorvastatin 40 mg nocte (every night), and syrup morphine PRN (as needed). Cardiac biomarkers and echocardiography (ECHO) were unremarkable, but her initial ECG (done 7 days prior) showed a strain pattern (Figure 1).

**Figure 2.**
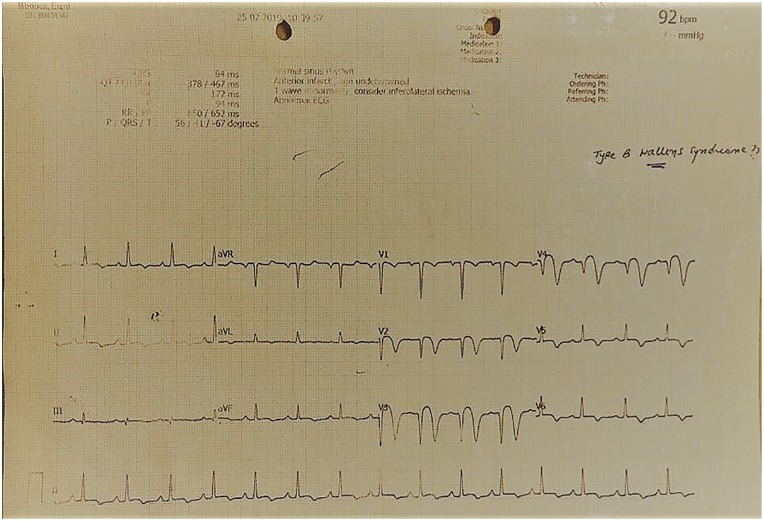
A 12-lead ECG displaying deeply inverted T-waves on leads V2 through V4.

She had stable vitals (blood pressure 133/74 mm Hg, pulse rate 75 beats/min, respiratory rate 19 breaths/min, temperature 36.8°C, and body mass index 22 kg/m^2^), and her systemic examination was unremarkable. Nonetheless, a 12-lead ECG done at our center revealed deeply inverted T-waves on leads V2 through V4 (Figure 2). A 2-dimensional ECHO showed anterior wall hypokinesia with preserved left ventricular (LV) systolic functions (ejection fraction 62%). Evaluation of valves and pericardium was unremarkable. Hematologic and biochemical tests were evident for a microcytic hypochromic anemia (hemoglobin 6.5 g/dL, mean corpuscular volume 73.2 fL, mean cell hemoglobin 21 fL, red cell distribution width 17.6%) otherwise normal. Cardiac biomarkers (troponin I and CK-MB) remained within acceptable limits. Furthermore, sickling test, glucose-6-phosphate-dehydrogenase deficiency, thrombophilia, and autoimmune (rheumatoid factor, antineutrophil cytoplasmic antibody, and antinuclear antibody) screenings revealed negative findings. Based on our extensive history, physical examination, and ECG features, we came up with a diagnosis of type B Wellens syndrome with impending anterior wall MI. Isosorbide mononitrate 10 mg bd (twice a day) and bisoprolol 5 mg od were added to the ongoing regime of acetylsalicylic acid, clopidogrel, and atorvastatin.

**Figure 3.**
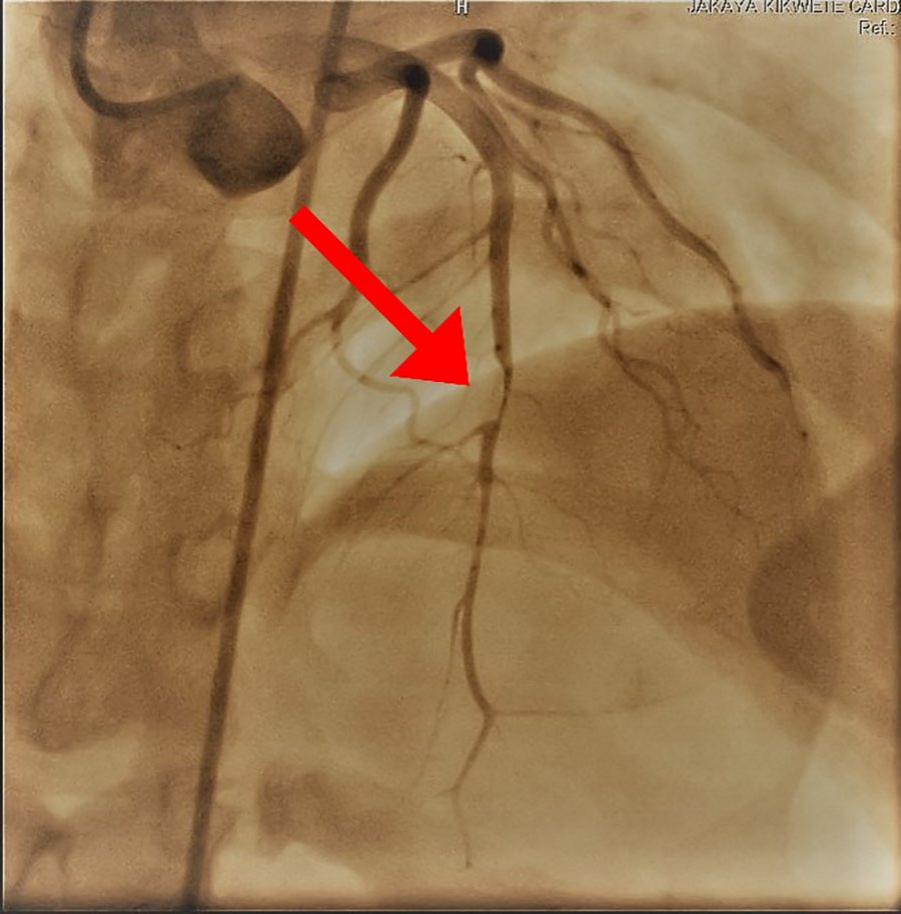
Coronary angiogram showing severe subocclusive (>95%) mid LAD stenosis.

**Figure 4.**
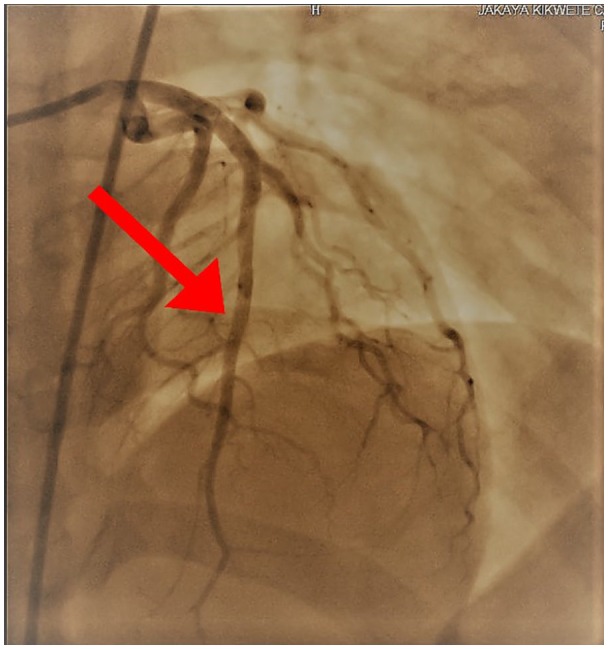
Coronary angiogram showing patent mid LAD after successful deployment of a resolute integrity stent.

**Figure 5.**
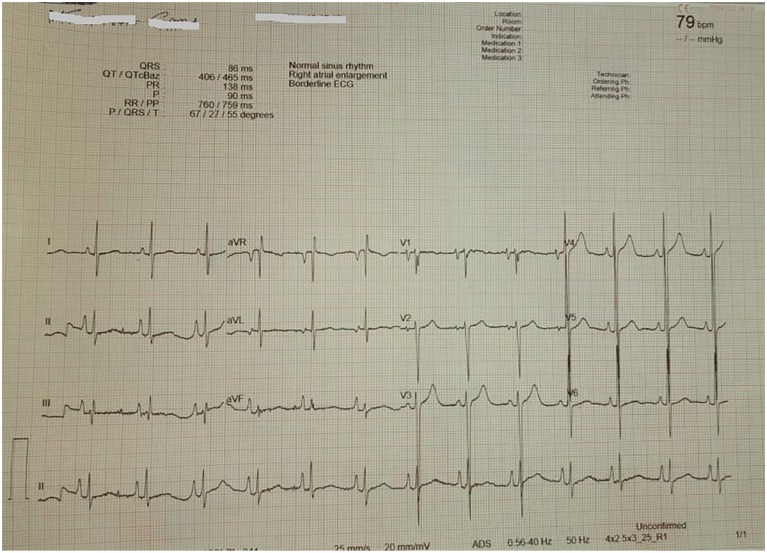
A 12-lead ECG during follow-up displaying unremarkable findings.

Due to the ongoing chest pain and pathognomonic ECG features, the patient was counseled and consented for an elective cardiac catheterization (on the 11th day since onset of chest pain), which revealed a nonthrombogenic severe subocclusive (>95%) mid-LAD stenosis with a TIMI I flow (Figure 3). The left main, circumflex, and right coronary arteries were unremarkable. Subsequently, angioplasty was performed successfully with a resolute integrity stent, and TIMI III flow was achieved (Figure 4). She was also transfused 3 units of packed red blood cells during hospitalization, and her control hemoglobin was 11.3 g/dL. During a brief coronary care unit stay, there was neither recurrence of chest pain nor hemodynamic instability; hence, the patient was transferred to the general cardiac ward in a fairly stable clinical state and was discharged after 13 days of hospitalization. Four weeks postdischarge, she was reviewed at our outpatient department, and although she was asymptomatic with an improved effort tolerance, a detailed ECHO revealed dyskinesia of the anterolateral wall with no evidence of LV aneurysm. Moreover, her ECG during follow-up was unremarkable (Figure 5). A magnetic resonance imaging for myocardial perfusion test was ordered but was not done due to patient’s financial constraints.

## Discussion

With an estimated contribution of up to 6% of all acute coronary events, young adults (<45 years) are experiencing an escalating burden and mortality attributable to CAD globally.^10-12^ Young patients presenting with chest pain may often appear deceptively well with no apparent risk factors or hemodynamic instability^10^; however, clinicians should strive to recognize it, exclude life-threatening causes, and intervene timely. Owing to the increasing prevalence of CAD risk factors in younger individuals, the traditionally known protection offered by young age is slowly fading and an increased disease burden will be apparent in the near future.^10,13^

Wellens syndrome is an ECG manifestation characterized by a pattern of deeply inverted or biphasic T-waves in leads V2 to V3, signifying a critical stenosis of the LAD coronary artery.^1-9^ Despite of its known diagnostic and prognostic value, recognition of Wellens syndrome remains a challenge to practitioners all over the world.^1-9^ As demonstrated in this case, a definitive diagnosis was reached on the eighth day since onset of chest pain. Arguably, this was so due to various reasons: (1) absence of pathognomonic features for Wellens syndrome in an initial ECG, (2) absence of traditional risk factors for CAD in this case that probably resulted in lack of aggressive workup including serial ECGs, and (3) poor understanding and low index of suspicion for Wellens syndrome among cardiovascular physicians working in the initial consultant referral hospital.

Cardiac enzymes may give false reassurance in patients with Wellens syndrome as they are frequently negative as it was in our case.^1-9^ Nevertheless, presence of positive cardiac biomarkers portends a poorer prognosis and warrants urgent catheterization as it has been associated with a higher incidence of total LAD occlusion.^6^ Furthermore, like in this case, stress test should be avoided in individuals with Wellens syndrome as it is associated with an increase in myocardial demand, which may induce MI, LV failure, and subsequent sudden death.^5^ As this case met the criteria for type B Wellens syndrome, which is comparatively less common (ie, 24%) than type A, it is worth mentioning that it is associated with higher fatality.^14^

The ability to accurately interpret abnormalities on ECG is a core competency for cardiovascular and emergency physicians that is central to the assessment of patients with potential cardiac ischemia. High-risk ECG patterns inform the diagnosis and management of patients with suspected acute coronary syndrome, but the failure to identify it or the impact of misinterpretation of the tracing is likely to result with a grievous consequence. Luckily, despite the delay in reaching the diagnosis, ECG performed at our institute revealed the typical Wellens syndrome features and was correctly interpreted by the attending cardiologist. Nonetheless, even after diagnosis realization coupled by ongoing chest pain, there was yet a further 3-day delay in taking the patient for revascularization (ie, elective case). As a consequence of cumulative delays both in diagnosis and intervention, and notwithstanding successful revascularization, echocardiographic assessment done 1 month later revealed persistent ischemic features suggestive of a significant myocardial injury.

Last, anemia in the setting of CAD has an established adverse prognostic value. It is associated with new onset and recurrence of acute coronary syndrome as well as increased risk for mortality both short and long term.^15,16^ Considering the presence of severe anemia coupled by absence of acute thrombotic evidence in coronary angiography, and assuming that there was a significant preexisting LAD disease, it is likely that a combination of such events triggered Wellens syndrome in the case presented.

To conclude, Wellens syndrome is a relatively common yet underrecognized preinfarction entity with an ill-fated MI progressive potential if unintervened. In view of this, physicians should be well aware of this subtle yet warning ECG sign as prompt recognition and a timely aggressive intervention is of paramount importance to curb the attributable morbidity and mortality. Moreover, presence of angina particularly in healthy young patients with no apparent risk factors for CAD should raise an index of suspicion for Wellens syndrome.
